# Pathophysiology of COVID-19: Critical Role of Hemostasis

**DOI:** 10.3389/fcimb.2022.896972

**Published:** 2022-06-03

**Authors:** Sonia Aparecida de Andrade, Daniel Alexandre de Souza, Amarylis Lins Torres, Cristiane Ferreira Graça de Lima, Matteo Celano Ebram, Rosa Maria Gaudioso Celano, Mirta Schattner, Ana Marisa Chudzinski-Tavassi

**Affiliations:** ^1^ Biopharmaceuticals Laboratory, Instituto Butantan, São Paulo, Brazil; ^2^ University of Taubaté (UNITAU), Taubaté, Brazil; ^3^ Laboratory of Experimental Thrombosis. Instituto de Medicina Experimental – CONICET -Academia Nacional de Medicina, Buenos Aires, Argentina; ^4^ Center of Excellence in New Target Discovery (CENTD), Instituto Butantan, São Paulo, Brazil; ^5^ Innovation and Development Laboratory, Instituto Butantan, São Paulo, São Paulo, Brazil

**Keywords:** COVID-19, SARS-CoV-2, hemostasis, coagulopathy, thrombus

## Abstract

The COVID-19 pandemic, caused by SARS-CoV-2, had its first cases identified in late 2019 and was considered a clinical pandemic in March 2020. In March 2022, more than 500 million people were infected and 6,2 million died as a result of this disease, increasingly associated with changes in human hemostasis, such as hypercoagulation. Numerous factors contribute to the hypercoagulable state, and endothelial dysfunction is the main one, since the activation of these cells can strongly activate platelets and the coagulation system. In addition, there is a dysregulation of the renin-angiotensin system due to the SARS-CoV-2 takeover of the angiotensin converting enzyme 2, resulting in a strong immune response that could further damage the endothelium. Thrombus formation in the pulmonary microvasculature structure in patients with COVID-19 is an important factor to determine the severity of the clinical picture and the outcome of this disease. This review describes the hemostatic changes that occur in SARS-CoV-2 infection, to further improve our understanding of pathogenic mechanisms and the interaction between endothelium dysfunction, kallikrein-kinins, renin angiotensin, and the Coagulation/fibrinolysis systems as underlying COVID-19 effectors. This knowledge is crucial for the development of new effective therapeutic approaches, attenuating the severity of SARS-CoV-2’s infection and to reduce the deaths.

## Introduction

COVID-19 is a disease caused by the severe acute respiratory syndrome coronavirus 2 (SARS-CoV-2), a virus of the Coronavidae family with an envelope and genome consisting of a single strand of positive-stranded RNA. COVID-19 had the first cases identified in Wuhan – China at the end of 2019, and was considered a pandemic by the World Health Organization (WHO) in March of 2020. To date, more than 500 million people have been infected, and over 6,2 million people have died worldwide as a result of this disease (Dong et al., 2020; WHO Coronavirus (COVID-19) Dashboard, 2021).

It is known that some people infected with the new coronavirus remain asymptomatic, but these people can still carry and transmit the virus (Johansson et al., 2021). Normally, COVID-19 is a mild illness associated with, fever, fatigue, cough, muscle aches, sore throat, loss of smell or taste, and other symptoms; However, some patients infected with SARS-CoV-2, may suffer different clinical manifestations, as severe respiratory syndrome and even death (Wiersinga et al., 2020). Turk et al. described three critical clinicobiological phases of SARS-associated coronavirus infections in humans: “asymptomatic/pre-symptomatic phase”, “respiratory phase with mild/moderate/severe symptoms” and “multi-systemic clinical syndrome with impaired/disproportionate and/or defective immunity” (Turk et al., 2020). Understanding the phases may be useful for clinical management and development of vaccines and/or specific drugs targeting the COVID-19 processes

The fatality rate of COVID-19 patients with diabetes was 7.3%, for patients with cardiovascular disease 10.5%, and for patients without any comorbidities 0.9% (Yang et al., 2020). Furthermore, when compared to according age groups, people with age 45 or higher are more likely to die from COVID-19 than the younger ones. Aging leads to structural and functional modifications of the vasculature, which may lead to endothelial dysfunction (Amraei and Rahimi, 2020). Endothelial cells play a major role in the pathogenesis of this disease, furthermore SARS-CoV-2 infection promotes changes in hemostasis; a recent study found that nearly 72% of non-survivors had evidence of hypercoagulability (Tang, 2020).

The major leading cause of mortality in patients with COVID-19 is respiratory failure from acute respiratory distress syndrome (ARDS) (Amraei and Rahimi, 2020). However, deaths resulting from COVID-19 are significantly associated with vascular injuries (Wu et al., 2021) and SARS-CoV-2 infection induces changes in the coagulation and fibrinolytic system (Leentjens et al., 2021). Such pathophysiological changes generate arterial and, mainly, venous thrombosis, especially in patients with severe symptoms. These thrombotic events occur more frequently in the lung, and both macro and microthrombi have been reported, the latter being usually not detectable by imaging, but only by post mortem autopsy (Asakura and Ogawa, 2021).

Post-mortem histopathological analysis of lung tissue from 38 patients with SARS-CoV-2 demonstrated, in most cases, the presence of fibrin- and platelet-rich thrombi in pulmonary arterioles, congested capillaries, and bleeding alveoli, often containing CD61+ megakaryocytes, and dense capillary foci, presumably resulting from angiogenesis (Carsana et al., 2020). In another post-mortem analysis in multiple tissues, there is a description of congestion and small vessel endotheliitis with accumulation of mononuclear/lymphocytic cells around the capillary endothelium in the heart, small intestine, kidney, liver and lung; in one case, caspase 3 immunostaining revealed the presence of apoptotic bodies in the endothelial cells lining the inner wall of inflamed blood vessels. As a consequence of this vascular disorder, after inflammation, congestion, thrombosis, hemorrhage, and endothelial cell death, the surrounding hypoxic tissues showed evidence of interstitial edema, destruction, inflammation, fibrosis, and vascular regeneration (Carsana et al., 2020; Varga et al., 2020). Deep vein thrombosis in the extremities was accompanied by evidence of recent thrombosis in the prostatic venous plexus in 6/7 cases by Wichmann and colleagues (Wichmann et al., 2020).

There are numerous factors that could contribute to the hypercoagulable state of COVID-19 patients, endothelial dysfunction being the main factor since endothelial cell activation can strongly activate platelets and the coagulation system (Yau et al., 2015). In addition, there is a dysregulation of the renin-angiotensin system due to SARS-CoV-2 takeover of the angiotensin converting enzyme 2 (ACE-2) resulting in a strong immune response that could further damage the endothelium (Amraei and Rahimi, 2020).

The pathophysiology of endothelial dysfunction and injury offers insights into COVID-19 associated mortality. Besides this, a process made up of three main steps which happen simultaneously in a fine orchestrated fashion – platelet aggregation, blood clotting and fibrinolysis. The integrity of the endothelium is also essential for the maintenance of hemostasis and any disturbances between any of these features can lead to hemorrhage or thrombosis (Maffei et al., 2015).

## Changes on the Endothelium

The infection and viral entry of SARS-CoV-2 into the cell is mediated by angiotensin ACE-2, transmembrane serine protease 2 (TMPRSS2) and cathepsin L, which cleaves the spike protein on the viral particle to allow engagement with ACE-2 (Jackson et al., 2021).

As it is known, some endothelial cells, especially in the lungs, highly express ACE-2 in its surface, making them a direct target for coronavirus infection (Hamming et al., 2004). ACE-2 is a type I transmembrane receptor with 3 domains, a single transmembrane domain, a cytoplasmic carboxyl domain, and a catalytic extracellular domain. The main physiological function of ACE-2 is in the regulation and metabolism of Renin-Angiotensin System peptides opposing the effects of angiotensin II, serving as a counter-regulatory mechanism to ACE. Usually, ACE-2 catalyzes polypeptides with preference for hydrolysis between proline and a hydrophobic aminoacid (Vickers et al., 2002).

ACE-2 generates Ang 1-9 peptide through cleavage of Ang I (1-10), while ACE converts Ang I into Ang II (Ang 1-8). ACE-2 also metabolizes Ang II (Ang 1-8) to generate Ang 1-7. The peptides generated by ACE-2 bind and activate the G-protein coupled receptor (GPCR). The receptor activation stimulates several major signaling pathways, including phospholipase A, which will further generate arachidonic acid (AA), phosphoinositide 3 kinase (PI3K)/AKT axis, which activates endothelial nitric oxide synthase (eNOS), and activation of phospholipase C, and an increase in intracellular calcium levels. These pathways regulate vasodilation, anti-fibrosis and anti-inflammatory responses in endothelial cells (Keidar et al., 2007; Heurich et al., 2014).

The internalization and shedding of ACE-2 could be mediated by the proteolytic activity of disintegrin and metalloprotease 17 (ADAM17) and transmembrane protease serine 2 (TMPRSS2) (Hoffmann et al., 2020). This serine competes with ADAM17 for ACE-2 processing and is found to promote SARS-CoV-2 entry by two mechanisms: ACE-2 cleavage, which promotes viral uptake and spike protein cleavage, which activates it for membrane fusion (Solinski et al., 2014).

**Figure 1:**
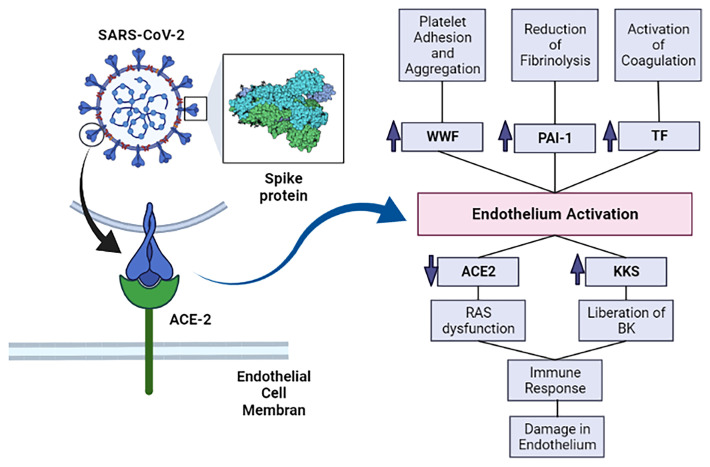
The SARS-CoV-2’s spike protein interacts with ACE-2 and allows SARS-CoV-2 infection. This infection causes activation of the endothelium and, subsequently, increases prothrombotic factors. The infection also enhances inflammation, which further damages the endothelium. ACE-2, angiotensin converting enzyme 2; KKS, Kallikrein-kinin system; RAS, renin-angiotensin system; BK, bradykinin; vWF, von Willebrand factor; PAI-1, plasminogen activator inhibitor–1; TF, Tissue factor. Image created in: biorender.com.

SARS-CoV-2 spike protein can trigger downregulation of ACE-2 expression in lung tissue and in cell culture, allowing higher binding of Ang II to AT1 receptors and ultimately vasoconstriction, enhanced inflammation and thrombosis (**Figure 1**) (Verdecchia et al., 2020).

There is strong evidence for a complex association between viral infections, inflammatory processes, and endothelial cells. The endothelium is a monolayer of endothelial cells that internally coat the blood vessels; its importance on hemostasis goes beyond acting as a barrier against blood loss (Wu, 1992). In normal conditions of hemostasis, the endothelium maintains a balance between the procoagulant and fibrinolytic factors, producing molecules that inhibit platelet aggregation, like nitric oxide (NO), anti-clotting molecules, like thrombomodulin, and other important substances for the fibrinolytic system, such as tissue plasminogen activator (tPA) (Wu and Thiagarajan, 1996).

When endothelial cells encounter pathogen associated molecular patterns (PAMPs) such as lipopolysaccharide, proinflammatory cytokines, interleukin 1 (IL-1), tumor necrosis factor (TNF), (IL-6) or damage associated molecular patterns (DAMPs) derived from dead or dying cells, they become activated. Endothelial cells’ activation can also occur as a direct cytopathic effect of viral infection (Libby and Lüscher, 2020). Although SARS-CoV-2 has been reported to directly infect vascular organoids (Monteil et al., 2020), and case studies reported endotheliitis in COVID-19 patients (Amraei and Rahimi, 2020), and endothelial infection in glomerular capillary loops and skin lesions it is still not clear whether vascular damage can be attributed to a systemic inflammatory response, or is a direct consequence of the viral infection and replication.

Endothelial activation is a key event that contributes to platelet activation, changes on hemostasis, and increase on vascular permeability, with decreased concentrations of anti-clotting molecules (Zhang et al., 2020). Once activated, the endothelium cells can express and exert tissue factor activity, amplifying the enzymatic activity of the coagulation cascade proteins and triggering thrombin generation and clot formation. These cells also release von Willebrand factor (vWF) from its Weibel-Palade bodies, stimulating platelet adhesion and aggregation (Lyons and Ginsburg, 1994). Associated with the prothrombotic effects of endothelial cells activation, there is also antifibrinolytic activity, mostly conformed by increased concentrations of plasminogen activator inhibitor–1 (PAI-1) (Wu and Thiagarajan, 1996).

SARS-CoV-2 infection of endothelium also triggers the secretion of the von Willebrand factor (vWF), PAI-1, soluble thrombomodulin, angiopoietin-2, an increase in endothelium-derived adhesion molecules (ICAM, VCAM-1 and P-selectin among others) expression, a decrease of endothelial progenitor cells circulation, as well as secretion of proinflammatory interleukins (IL-1, IL6) (Zhang et al., 2020). IL-1 can induce the production of more IL-1, IL-6 and other proinflammatory cytokines, and this overproduction is named a “cytokine storm”. IL-1 stimulation also reduces VE-Cadherin, which maintains the integrity of the endothelium (Libby and Lüscher, 2020).

Neutrophils have also been shown to contribute to endothelial damage in COVID-19. Under the influence of the proinflammatory state, neutrophils in contact with endothelial cells can release enzymes such as myeloperoxidase (MPO), proteinase 3 (PR3), neutrophil elastase (NE) and cathepsin G (CG) (Qi et al., 2017). Long-term exposure of the endothelium to these enzymes can lead to disruption of the endothelial barrier and cell apoptosis, exposing the subendothelium to platelets and leukocytes (Caillon et al., 2021).

Autopsy studies have shown important endothelial damage of the lung microvasculature, including loss of “tight junctions”, separation of the endothelium from its basal membrane and apoptosis of endothelial cells (O'Sullivan et al., 2020; Zhang et al., 2020). These apoptotic cells express the ACE-2 receptor, and analysis by electronic microscope found the presence of SARS-CoV-2 virions in these cells (Carsana et al., 2020).

## Alterations in the Number and Function of Platelets

Platelets are anucleate cell fragments derived from the bone marrow and lung megakaryocytes. When vascular lesion occurs, platelets quickly adhere to the exposed subendothelium. In conditions of high shear tension, which is present in arterial vessels, adhesion is mainly due to the binding of the vWF to glycoprotein Ib (GPIb) present on the platelet’s surface. On the other hand, in conditions of low shear stress, which occur in the venous part of the circulatory system, platelet adhesion mainly happens through the interaction of collagen with the glycoprotein VI (GPVI) or the integrin alpha(2) beta(1) (Ruggeri et al., 2006). As a consequence of this adhesion, platelets become activated, there is a change in the organization of their cytoskeleton which changes its shape, from discoidal to irregular, with generation of filopodia. Besides, there is exocytosis of granules, which mainly contain agonists, like ADP and serotonin. These molecules interact with specific receptors on the platelet surface, and as a result activate more platelets. Platelet activation favors the binding of fibrinogen with integrin alpha (IIb)beta(3) and this binding is essential, as it allows a connection between adjacent platelets and platelet aggregation (Peter et al., 1998; Jurk and Kehrel, 2005).

Platelet’s morphological and biochemical changes are relevant to COVID-19 pathophysiology. There is evidence that many pathways of platelet activation are intensified after infection with SARS-CoV-2, either by an indirect path through the action of inflammatory cytokines and endothelial damage or directly through viral infection. Moreover, it has been demonstrated that SARS-CoV-2 is capable to infect and replicate in megakaryocytes in the bone marrow and in the lung. Whether these megakaryocytes produce platelets carrying virions is still not known (Barrett et al., 2021). Overall, cytokine storm, thrombin generation, endothelium dysfunction, activation of (C3a) complement, increase in viscosity and hypoxia are considered the main reasons for platelet activation and aggregation caused by SARS-CoV-2.

Generally speaking, thrombocytopenia is frequent in severely ill patients, being associated with bad clinical prognosis and, also, death. Many COVID-19 patients, mainly those in intensive care, display thrombocytopenia, associated with the worst clinical outcomes. A meta-analysis of 31 studies with 7163 participants observed thrombocytopenia on stern cases, and this was associated with a 3-fold risk of developing severe COVID-19 (Jiang et al., 2020). Some mechanisms proposed as the main pathways leading to thrombocytopenia in COVID-19 are: impaired platelet production, immune depletion and trapping within growing thrombus and peripheral embolization (Zhang et al., 2020).

Some markers of platelet activity, such as the maturity and size of the platelets are significantly associated with severe COVID-19 cases and its lethality (Lyons and Ginsburg, 1994). Besides this, it has been shown that there is a difference in the transcriptome of platelets isolated from COVID-19 patients in comparison to non-infected platelets. The platelet phenotype is more immature, and changes occur on metabolic paths, including oxidative phosphorylation and glycolysis (Grove et al., 2009). Platelets do not act alone, as they amplify extracellular vesicle emission and tissue factor expression in monocytes through the interaction of P-selectin with P-selectin glycoprotein ligand-1 which is exposed on the surface of monocytes and neutrophils (Zhang et al., 2020). Communication with dysfunctional endothelium and neutrophils are key points for neutrophil and platelet activation (Hottz et al., 2020). In fact, it has been recently shown that alterations in circulating neutrophils rather than in the endothelium, are major contributors to the increased thrombotic diathesis in the hearts of COVID-19 patients (Johnson et al., 2022).

Other important playmakers in this process include extracellular vesicles (EV), released normally by activated leukocytes, platelets and endothelium. They carry and signal several physiological phenomena, such as inflammation, coagulation, and are related to thrombosis in some cardiovascular diseases (Ridger et al., 2017). They are important to coagulation, since they promote thrombin formation by exposing tissue factor and negatively charged phospholipids. Additionally, they promote thromboinflammation indirectly, stimulating the release of pro-inflammatory endothelial cytokines, inteleukin-8 (L-8), IL-6, and monocyte chemotactic protein 1 (MCP-1), inducing endothelial activation, expression of cyto-adhesins, and diapedesis. Some previous papers showed increased circulating EV secreted by platelets and leukocytes in patients with COVID-19 (Zaid et al., 2020; Krishnamachary et al., 2021). This is a topic that has not been fully explored and needs further studies to improve our scientific understanding.

Knowledge gathered until now has shown that some platelet activation mechanisms contribute to the thrombotic effects of COVID-19, hence platelet changes are relevant to the development and symptoms of this illness.

## Alterations in Coagulation

The coagulation cascade is made up of a series of reactions that culminate in the formation of a fibrin clot, which contributes to prevent bleeding in a vascular lesion. The formation of fibrin depends on the action of thrombin, that cleaves fibrinogen, releasing A and B fibrinopeptides, so as to form the fibrin monomers that polymerize, forming an insoluble net of fibrin. Besides this, thrombin activates FXIII, which then connects the fibrin fibrils through lysine residues, contributing to a greater stability of the clot (Siebenlist et al., 2001). Thrombin is generated from its inactive precursor – prothrombin - through the action of FXa.

It is possible to didactically divide the coagulation cascade into two distinct pathways that lead to the activation of FX: the extrinsic and intrinsic pathways. The extrinsic pathway starts when the lesion of the blood vessel exposes the tissue factor, which is made up of cells such as fibroblasts, and this contact with the intravascular medium, together with the FVIIa, activates FX. Through the intrinsic pathway, however, the contact of blood with the negative surfaces leads to the activation of FXII (contact activation) which starts a cascade that leads to the activation of FX (Macfarlane, 1964).

Activation of FXII to FXIIa by contact with negatively charged surfaces also starts the contact system. The contact system is part of the innate immune system and inflammatory response mechanism against pathogens. Factor XII, prekallikrein (PK) and high-molecular weight kininogen (HK) participate in the coagulation cascade as well as the contact system, having pivotal roles in the latter. This system can be activated by DNA, RNA, PAMPs, DAMPs, neutrophil extracellular traps and even activated platelets (Ito, 2014). In addition, both eukaryotic and prokaryotic RNA serve as activators of FXII and FXI, thus leading to activation of the contact system and inducing immunothrombosis (Kannemeier et al., 2007).

The kallikrein-kinin system (KSS) is also entangled in the mechanisms that maintain hemostasis. Although the contact system and the kallikrein-kinin system overlap, the activation of either has different implications. Activation of the KKS leads to the liberation of bradykinin (BK), a vasoactive peptide that plays a pivotal role in inflammation. After binding to bradykinin receptor 2 (B2R), BK activates a signaling pathway resulting in pain, fever, edema, hypotension, vasodilatation and increased vascular permeability (Oehmcke-Hecht and Köhler, 2018). It’s important to mention that BK also stimulates the production of IL-1, TNF-alpha and reactive oxygen substances, which, in turn, cause endothelial disruption (Tiffany and Burch, 1989).

**Figure 2:**
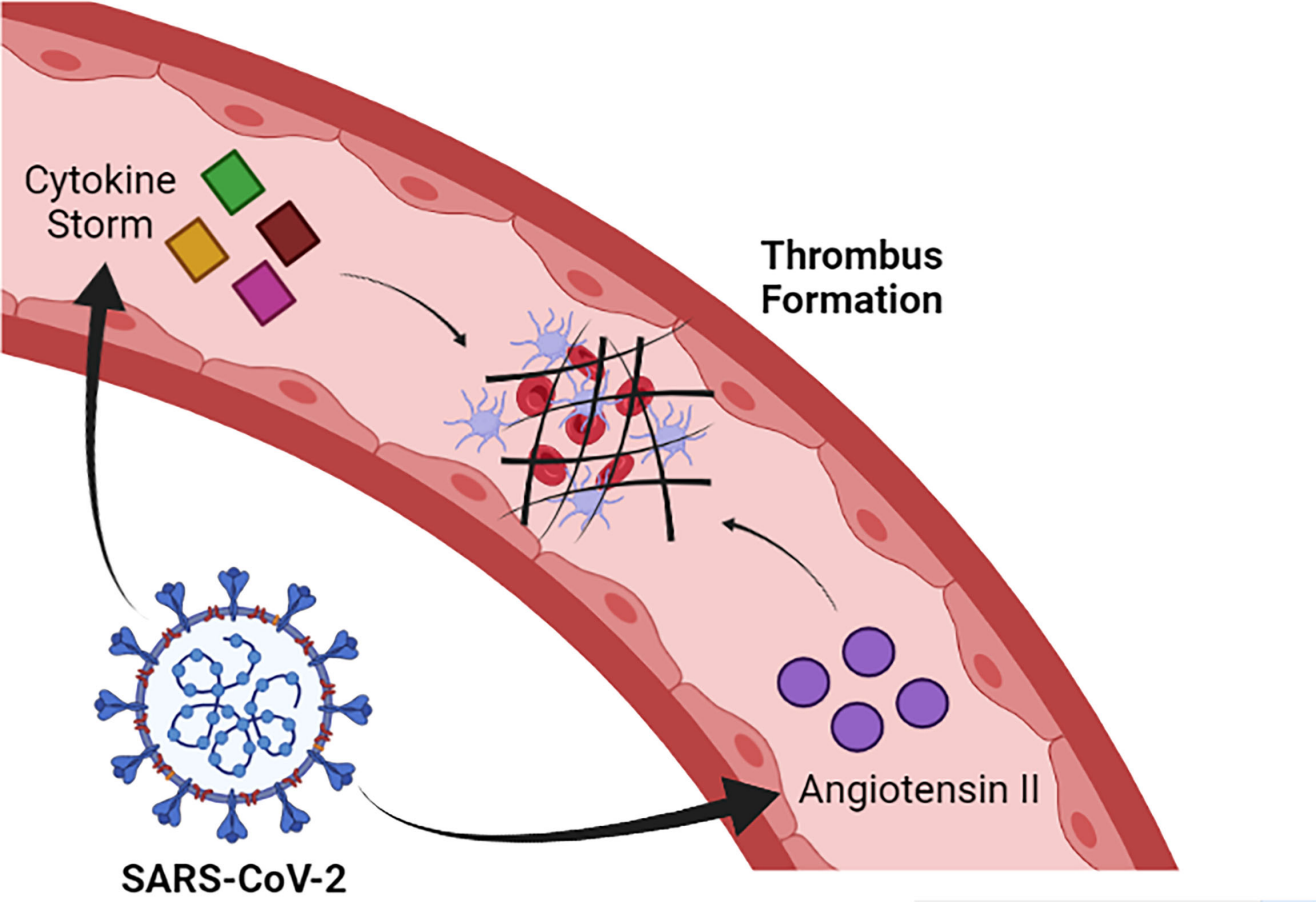
SARS-CoV-2 induces cytokine storm formation and increased levels of angiotensin II. Such alterations contribute to the activation of the coagulation cascade and, consequently, to thrombus formation. Image created in: Biorender.com.

SARS-CoV-2 infection induces alteration in coagulation and, in severe cases, can trigger disseminated intravascular coagulation (DIC) and thrombotic events, especially in the pulmonary microvasculature, which contributes to the evolution of dysfunction in this organ (Kannemeier et al., 2007). The mechanisms that lead to this clinical manifestation are not fully understood, but it is likely that the intense release of pro-inflammatory cytokines contributes to trigger the activation of the coagulation cascade. In this sense, there is a large release of IL-1 and IL-6 and TNF-alpha during the cytokine storm induced by SARS-CoV-2 infection (Han et al., 2020). IL-6 has an especially well described role in helping to activate coagulation by promoting the synthesis of fibrinogen, FVIII and tissue factor (TF) (Stouthard et al., 1996). Additionally, SARS-CoV-2 infection reduces the amount of ACE-2, which results in increased levels of angiotensin II. Elevated levels of angiotensin II favor the activation of coagulation and inhibition of the fibrinolytic system, which favors the prothrombotic state in COVID-19 (**Figure 2**) (Lazzaroni et al., 2021; Salabei et al., 2021).

It is observed that coagulation tests such as activated partial thromboplastin time (aPTT) and prothrombin time (PT) tend to be higher in symptomatic COVID-19 patients than in healthy individuals (Zhu et al., 2021; Luo et al., 2021). Although several studies indicate that aPTT, and especially PT, are also considerably higher in patients who died than in people who had less severe cases of COVID-19, a meta-analysis indicated that the results described in the literature are very heterogeneous, requiring caution and more data to establish a clear relationship between the severity of COVID-19 and PT and aPTT values (Lin et al., 2021).

In patients with COVID-19, especially those with greater severity and who died, they presented increased levels of fibrinogen concentration. This finding is quite different from what is normally observed in cases of consumptive coagulopathy associated with sepsis, in which a fall in fibrinogen levels is associated with mortality (van Vught et al., 2021). Furthermore, sepsis-induced coagulopathy usually has a much more marked prolongation of global clotting times than in COVID-19 cases (Lin et al., 2021). Thus, changes in the coagulation cascade caused by COVID-19 seem to be quite distinct of this disease.

Another alteration typically observed in COVID-19 patients is an increase in the activity and amount of vWF. The vWF is a circulating adhesive glycoprotein that promotes platelet aggregation, contributes to blood coagulation forming a complex with factor VIII, regulates angiogenesis, and vascular permeability. vWF levels are elevated in inflammation, aging, diabetes and other diseases associated with endothelial dysfunction (Amraei and Rahimi, 2020). Besides this, in patients with severe cases, there is a downregulation in the activity of ADAMTS-13 through different mechanisms, with a small reduction of these activities, and an increase on the level of their inhibitors. The consumption of the vWF high molecular weight multimers (HMWM—vWF) is common in patients that require intensive therapy (Philippe et al., 2021). In contrast with other types of sepsis, an increase in the vWF/ADAMTS-13 ratio was observed, and a significant inverse correlation between vWF : Ag levels and ADAMTS-13 activity (Ward et al., 2021). This unbalance between substrate and enzyme in a tangential stress condition is probably even more noticeable in the lung microvasculature, the site where endothelial damage becomes more evident with greater formation of micro-thrombus. Moreover, it is known that the plasmatic distribution of the vWF multimers in these patients is similar to those found in acute thrombotic thrombocytopenic purpura patients (Ward et al., 2021).

## Alterations in the Fibrinolytic System and Anticoagulation

The fibrinolytic system is an important defense against intravascular thrombosis, and there is substantial evidence that the imbalance of this system is involved in the pathophysiology of cardiovascular ischemic events and endothelial dysfunction. The imbalance is driven, at least in part, by inappropriate activity of the renin–angiotensin system, which interacts with the fibrinolytic system at the level of the endothelium (Chapin and Hajjar, 2015).

Although the formation of the fibrin network is essential to prevent blood leakage, a system is needed to prevent its unrestrained formation, which can obstruct the vessels. This function is mainly performed by the fibrinolytic system, whose main component is plasmin, a serine protease that degrades fibrin, generating soluble fibrin degradation products (FDP).

Plasmin is generated from its inactive precursor, plasminogen, which can be activated by tissue tPA or by urokinase plasminogen activator (uPA). Once initiated, fibrinolysis is accelerated by a positive feedback mechanism. These activators can be inhibited by PAI-1, whereas plasmin can be directly inhibited by alpha2-antiplasmin (Mutch et al., 2007). Endothelial cells and vascular smooth muscle cells are the main source of tPA and PAI-1, controlling fibrinolysis locally.

The coagulation cascade can still be inhibited in order to avoid its excessive activation. The most relevant coagulation inhibitors are tissue factor protease inhibitor (TFPI), protein S, thrombomodulin, protein C and antithrombin. The mechanisms of action of these inhibitors are varied. For example, antithrombin directly inhibits thrombin, which is facilitated by the presence of heparin or heparan sulfate, whereas TFPI, produced by endothelial cells, inhibits both the tissue factor/FVIIa complex and FXa. Also, the C1 esterase inhibitor (C1-INH), natural regulator of the complement, kallikrein-kinin, contact and fibrinolytic system, is being investigated for treatment of COVID-19, targeting multiple systems involved in the disease (Adesanya et al., 2021).

In critically ill COVID-19 patients, studies have demonstrated reduction of natural anticoagulant systems, with decreased serum concentration of antithrombin and protein C, which can contribute to the hypercoagulability state that characterizes SARS-CoV-2 pathophysiology. It is known that plasma natural coagulation is decreased in patients with sepsis or DIC, and is also associated with disease severity (Zhang et al., 2020). A hypofibrinolytic state, additionally, has been observed, reflecting changes in the fibrinolytic system, with increased TFPI concentration (Caciola et al., 2021). As a consequence, the thrombi formation becomes easier, mainly inside the pulmonary microvasculature. More studies are needed to evaluate other parameters of natural coagulation, in order to determine the importance of these changes on the whole picture of COVID-19 disease.

In COVID-19 it is also observed, especially in more severe cases, an increase in the concentration of D-dimers, molecules produced due to fibrin degradation. Markedly increased D-dimers were detected early on in patients with COVID-19 (Huang et al., 2020). Elevation in D-dimer levels was associated with poor disease prognosis, and its dosage was extensively performed globally as a laboratory test in patients’ admission (Rostami and Hassan, 2020; Yu et al., 2020). Different papers try to suggest D-dimer cut-off levels as a prognostic indicator (Favaloro and Thachil, 2020; Zhou et al., 2020). Despite this, there is still controversy regarding the mechanism that causes the increase in the levels of D-dimers, and it is possible that this effect is a result only of the increase in the amount of fibrin formed in SARS-CoV-2 infection (Lazzaroni et al., 2021).

## Vascular Changes in the Lung

The pulmonary vasculature is responsible for the perfusion of these organs and is essential for proper hemostasis (Lammers et al., 2021). Histopathological studies demonstrating the structural and vascular changes in the lungs caused by COVID-19 are still limited. However, it has been reported that patients who died from COVID-19 often present hemorrhage, deposition of fibrin and, most importantly, formation of microthrombi in the pulmonary vasculature. In this sense, the formation of microthrombi in pulmonary capillaries occurs with greater intensity in COVID-19 than in influenza and is a factor that reduces respiratory efficiency by contributing to increase the dead space in ventilation (Pannone et al., 2021).

*Post-mortem* analysis also suggests that severe SARS-CoV-2 infection increases angiogenesis in the lung more intensely than seen with other respiratory infections such as influenza. This effect can be explained, at least in part, by the infiltration of pro-inflammatory cells, mainly macrophages, which are also capable of releasing pro-angiogenic compounds. The excessive proliferation of blood vessels in severe cases of COVID-19 abnormally increases perfusion, and is thus a factor that reduces the ratio of ventilation to perfusion in the lungs, contributing to hypoxaemia (Osuchowski et al., 2021). The formation of pulmonary edema is also associated with severe COVID-19, which occurs as a result of increased permeability of alveolar blood vessels in response to the interaction between kinins and their receptors on endothelial cells (Pérez-Mies et al., 2021).

COVID-19, especially in severe cases, was still associated with bleeding higher risk of hemorrhage, deep vein thrombosis and, especially, pulmonary embolism than individuals without this disease. These effects of COVID-19 remained significant even when adjusting for correlations for comorbidities and other risk factors, such as the advanced age of patients. Furthermore, it was observed that these effects did not appear to be minimized in individuals undergoing chronic anticoagulant therapy (Katsoularis et al., 2022).

## Conclusions

The clinical manifestations of COVID-19, especially in severe cases, are intrinsically related to hemostatic disorders caused directly or indirectly by SARS-CoV-2 infection. In general, COVID-19 promotes the occurrence of a prothrombotic state in the patient, which contributes to the obstruction of blood vessels. Thrombus formation in COVID-19 is mainly favored by the establishment of an inflammatory state that leads to endothelial activation, which, in turn, contributes to the excessive platelet aggregate formation, activation of the coagulation cascade and inhibition of the fibrinolytic system. In addition, the acquisition of ACE-2 influences the renin-angiotensin and kallikrein-kinin systems toward a prothrombotic state. The hemostatic changes resulting from COVID-19 are mainly manifested in the pulmonary microvasculature, being an important factor for the impairment of respiratory function observed in patients, especially those with more severe conditions.

## Future Directions

The detailed investigation of the mechanisms related to these alterations can contribute not only to a better understanding of COVID-19 pathophysiological mechanisms but can also indicate new directions for possible treatments in the infection by SARS-CoV-2 and better monitoring of hospitalized patients. Therefore, this review aims to further improve the understanding of the pathophysiology of COVID-19 by providing a detailed description of the molecular mechanisms involved in human hemostasis alterations after SARS-CoV-2 infection. We believe that better understanding of the CS, KKS, RAS and/or the Coagulation/Fibrinolysis systems, as well as how the interactions occur between them, and their consequences in the hemostasis, can contribute to a better understanding of the thrombotic state, observed in COVID-19. The knowledge of these mechanisms is crucial for the better disease understanding and could lead to therapies that modulate the human hemostasis, attenuating or inhibiting vessel obstruction, especially of the pulmonary microvasculature, due to the formation of thrombi.

## Author Contributions

SA, DS, AT, ME, RC, CL, MS, and AC-T conceived the manuscript, reviewed the literature, and wrote the manuscript. All authors contributed to the article and approved the submitted version.

## Funding

Financial support: CNPq, CAPES, FAPESP and Fundação Butantan.

## Conflict of Interest

The authors declare that the research was conducted in the absence of any commercial or financial relationships that could be construed as a potential conflict of interest.

## Publisher’s Note

All claims expressed in this article are solely those of the authors and do not necessarily represent those of their affiliated organizations, or those of the publisher, the editors and the reviewers. Any product that may be evaluated in this article, or claim that may be made by its manufacturer, is not guaranteed or endorsed by the publisher.
